# The Usefulness of Serum Brain Damage Biomarkers in Detection and Evaluation of Hypoxic Ischemic Encephalopathy in Calves with Perinatal Asphyxia

**DOI:** 10.3390/ani12223223

**Published:** 2022-11-21

**Authors:** Mahmut Ok, Amir Naseri, Mehmet Burak Ates, Merve Ider, Kamil Uney, Mutlu Sevinc, Fatih Hatipoglu, Ramazan Yildiz, Alper Erturk, Nuri Baspinar, Suleyman Serhat Iyigun

**Affiliations:** 1 Department of Internal Medicine, Faculty of Veterinary Medicine, Selcuk University, Konya 42250, Türkiye; 2 Department of Pathology, Faculty of Veterinary Medicine, Selcuk University, Konya 42250, Türkiye; 3 Department of Pharmacology and Toxicology, Faculty of Veterinary Medicine, Selcuk University, Konya 42250, Türkiye; 4 Department of Internal Medicine, Faculty of Veterinary Medicine, Burdur Mehmet Akif Ersoy University, Burdur 15030, Türkiye; 5 Department of Internal Medicine, Faculty of Veterinary Medicine, Hatay Mustafa Kemal University, Hatay 31060, Türkiye; 6 Department of Biochemistry, Faculty of Veterinary Medicine, Selcuk University, Konya 42250, Türkiye

**Keywords:** perinatal asphyxia, calf, brain damage, serum biomarkers, mortality

## Abstract

**Simple Summary:**

The objective of the present study was to determine hypoxic brain damage in calves with perinatal asphyxia using brain-specific damage biomarkers. Ten healthy calves and 25 calves with perinatal asphyxia were enrolled in the study. Consciousness evaluation and laboratory analyses were performed at admission, 24, 48, and 72 h. Serum concentrations of brain-related biomarkers were measured to assess brain injury. Moreover, histopathological and immunohistochemical examinations of the brain tissue were performed in 13 nonsurvivor calves. The consciousness level of the calves with asphyxia was significantly lower than the healthy calves. Mix metabolic-respiratory acidosis and hypoxemia were detected in calves with asphyxia. Serum UCHL1 and S100B concentrations were significantly increased, and NSE, ACTA, ADM, and CK-B were decreased in calves with asphyxia. Histopathological and immunohistochemical examination in nonsurvivor calves confirmed the development of mild to severe hypoxic-ischemic encephalopathy. In conclusion, asphyxia causes hypoxic ischemic encephalopathy in perinatal calves. UCHL1 and S100B were found to be useful markers of hypoxic-ischemic encephalopathy in calves with perinatal asphyxia. Neurological status scores and some blood gas parameters were helpful in mortality prediction.

**Abstract:**

The purpose of the present study was to determine hypoxic brain damage in calves with perinatal asphyxia using brain-specific damage biomarkers. Ten healthy and 25 calves with perinatal asphyxia were enrolled in the study. Clinical examination, neurological status score, and laboratory analysis were performed at admission, 24, 48, and 72 h. Serum concentrations of ubiquitin carboxy-terminal hydrolysis 1 (UCHL1), calcium-binding protein B (S100B), adrenomodullin (ADM), activitin A (ACTA), neuron-specific enolase (NSE), glial fibrillary acidic protein (GFAP) and creatine kinase-brain (CK-B) were measured. Histopathological and immunohistochemical examinations of the brain tissue were performed in 13 nonsurvivor calves. The neurological status score of the calves with asphyxia was significantly (*p* < 0.05) lower. Mix metabolic-respiratory acidosis and hypoxemia were detected in calves with asphyxia. Serum UCHL1 and S100B were significantly (*p* < 0.05) increased, and NSE, ACTA, ADM, and CK-B were decreased (*p* < 0.05) in calves with asphyxia. Histopathological and immunohistochemical examinations confirmed the development of mild to severe hypoxic-ischemic encephalopathy. In conclusion, asphyxia and hypoxemia caused hypoxic-ischemic encephalopathy in perinatal calves. UCHL1 and S100B concentrations were found to be useful markers for the determination of hypoxic-ischemic encephalopathy in calves with perinatal asphyxia. Neurological status scores and some blood gas parameters were helpful in mortality prediction.

## 1. Introduction

Perinatal asphyxia, which is one of the important causes of non-infectious mortality in newborn calves, is characterized by hypoxia, hypercapnia, and acidosis [1,2,3]. Brain injury due to decreased blood flow to the brain tissue (ischemia) and oxygen deficiency (hypoxemia) is called hypoxic-ischemic encephalopathy (HIE) [4,5]. The morbidity range of HIE is between 4–57%, and it is responsible for 20–50% of perinatal mortality in human infants [6].

In recent years, it has been demonstrated that significant changes occur in ubiquitin C-terminal hydrolase 1 (UCHL1), calcium-binding protein B (S100B), adrenomodullin (ADM), activin A (ACTA), neuron-specific enolase (NSE), glial fibrillary acidic protein (GFAP) and creatine kinase-brain (CK-B) in hypoxia-induced brain damage in infants with neonatal asphyxia, and these markers have a significant contribution to the early detection of brain damage [7,8,9,10]. UCHL1, which is found in neurons and neuroendocrine cells, increases in blood and cerebrospinal fluid during brain barrier permeability damage and neuronal injury [11]. It has been reported that UCHL1 concentrations are higher in foals with hypoxic-ischemic encephalopathy compared to healthy foals, and it can be used as a diagnostic marker to determine HIE-related brain damage in foals [12]. S100B, an acidic calcium-binding peptide, was found to have increased concentrations in neurons and glial damage [13]. It has been found to be a reliable marker for assessing brain damage development in infants with perinatal asphyxia [14]. ADM, a hypotensive vasodilator peptide, is synthesized in the organism as ADM preproadrenomodulin. Previous studies stated that it could be used in the detection of neonatal neurological disorders since it provides cerebral vascular regulation in perinatal hypoxia [15]. Activin A is a protein that plays important biological effects in mesoderm induction, neuron cell differentiation, hematopoiesis, and reproductive physiology. It is suggested that activin A has a neuroprotective role in preterm infants with cerebellar hypoxia [16]. NSE is an isoenzyme that is found in neurons and neuroendocrine tissues. It has been reported that the NSE concentrations increased significantly in infants with neonatal asphyxia [17], and an increase in NSE concentration could be useful in the evaluation of neuronal damage severity and prognosis [18,19]. GFAP is a monomeric filament protein synthesized in astroglial cells [20]. GFAP has been shown to be a useful biomarker in the diagnosis and prognosis of neonatal hypoxic-ischemic encephalopathy [20]. CK-B is an isoenzyme found in neurons and astrocytes. It has been reported that CK-B enzyme activity increases significantly in infants with perinatal asphyxia [21].

Studies on perinatal asphyxia stated that HIE develops in humans, rats, horses, and pigs, and the usefulness of brain damage biomarkers in the diagnosis of HIE. However, no study has been found on calves. Therefore, the main hypothesis of the present study was to determine HIE development in calves with perinatal asphyxia and the utility of brain-specific biomarkers in detecting brain damage. This study, it was aimed to explore brain damage by histopathological methods to evaluate the neurological status and the utility of brain-specific damage biomarkers in the detection of possible brain tissue damage in calves with perinatal asphyxia.

## 2. Materials and Methods

The study protocol was approved by the Institutional Ethics Committee of the Faculty of Veterinary Medicine, Selcuk University (No. 2019/56) and conducted from November 2020 to September 2022.

### 2.1. Healthy Calves

A convenience sample of 10 healthy calves (7 Holstein, 3 Brown Swiss) with gestation age > 280 days, weighing 41 kg (39–44), and within the first 6–24 h after parturition were enrolled in the study. Calves were determined to be healthy based on clinical examinations and laboratory findings [3,22,23,24]. The calves were naturally born in faculty farm and were able to stand within 1 h and were fed 2 L of colostrum within the first 2 h of life. Calves from dystocia, prematurity, congenital abnormalities, and infection suspicion were excluded from the study.

### 2.2. Calves with Asphyxia

A convenience sample of 25 calves (19 Holstein, 4 Simmental, and 2 Brown Swiss) with gestation age >280 days and weighing 46 kg (43–54) admitted to the Large Animal Hospital of the Faculty of Veterinary Medicine, Selcuk University were enrolled in the study. All the calves included in the study were born from dystocia and within the first 6–24 h after parturition. They did not receive colostrum or any veterinary intervention. Calves with congenital abnormalities, prematurity, infection suspicion, and diarrhea were excluded from the study. All calves with asphyxia received standard supportive treatment, which included oxygen therapy and a feeding protocol following admission to the neonatal intensive care unit [3,25,26].

### 2.3. Clinical Examination and Neurological Status

Clinical examinations (evaluation of hydration status, palpable lymph nodes, mucous membranes, measurements of heart and respiratory rate, heart and lung auscultation) of all calves were performed at admission, 24, 48, and 72 h. Moreover, to evaluate the level of consciousness in calves, a simplified modified Glasgow coma scale (mGCS) based on motor activity, brain stem reflexes, and level of consciousness was performed. In this rating system, each category is scored between 1–3, with 1 being indicative of more severe dysfunction. The scores from each category are added together to establish a neurological status and categorized as grave, 8–13; guarded, 14–19; good, 20–24 (Table 1).

### 2.4. Criteria for Definition of Respiratory Distress Syndrome (RDS)

The criteria for RDS were respiratory acidosis, hypoxia (PaO_2_ < 60 mmHg), hypercapnia (PaCO_2_ > 45 mmHg), tachypnea (respiratory rate > 45/min), abdominal respiration, and hyperlactatemia (>6 mmol/L) [3,25,26]. Among these parameters, the presence of at least two criteria along with PaO_2_ < 60 mmHg was taken into consideration [1,25,27].

### 2.5. Collection of Blood Samples

Blood samples were collected from the calves at the time of admission, 24, 48, and 72 h. Blood samples for complete blood count (CBC) and serum were taken from the jugular vein and for blood gas measurement from auricular arteries. Tubes with K_3_EDTA and non-anticoagulant tubes were used for CBC and serum collection, respectively. Sodium heparin-containing plastic syringes were used for blood gas measurement. Blood samples taken for biochemical analyses were kept at room temperature for 15 min, then centrifuged at 2000× *g* for 10 min. Sera were removed and stored at −80 °C. Blood gas measurements were performed within 5 to 10 min of collection.

#### 2.5.1. Blood Gas and Chemistry Analysis

Arterial blood pH, partial oxygen pressure (PaO_2_), partial carbon dioxide pressure (PaCO_2_), oxygen saturation (SO_2_), lactate, glucose, sodium (Na), potassium (K), calcium (Ca), chlorine (Cl), bicarbonate (HCO_3_), and base deficit (BE) measurements were performed using an automatic blood gas analyzer (ABL 90 Flex, Radiometer, Brea, CA, USA).

#### 2.5.2. Complete Blood Count Analysis

Total leukocyte (WBC), erythrocyte (RBC), hematocrit (HCT), hemoglobin (Hb), and thrombocyte (THR) measurements were performed using an automatic cell counter (MS4e, Melet Schlosing Laboratories, Osny, France).

#### 2.5.3. Evaluation of Brain-Related Biomarkers

Serum UCHL1, S100B (Bioassay Technology Laboratory, Shanghai, China), ADM, ACTA, NSE, GFAP (MyBioSource, San Diego, CA, USA), and CK-B (ELK Biotechnology Co., Ltd., Wuhan, China) concentrations were measured with commercial bovine-specific ELISA test kits in accordance with the manufacturer’s instructions. Bovine UCHL1 commercial ELISA kit (Bioassay Technology Laboratory, Shanghai, China, Lot: 202110012), bovine S100B commercial ELISA kit (Bioassay Technology Laboratory, Shanghai, China, Lot: 202110012), bovine ADM commercial ELISA kit (MyBioSource^®^, San Diego, CA, USA, Lot: 38400921), bovine ACTA commercial ELISA kit (MyBioSource^®^, San Diego, CA, USA, Lot: 20211022C), bovine NSE commercial ELISA kit (MyBioSource^®^, San Diego, CA, USA, Lot: 36379821), bovine GFAP commercial sandwich ELISA kit (MyBio-Source^®^, San Diego, CA, USA, Lot: 34358721), and bovine CK-B commercial ELISA kit (ELK Biotechnology, Wuhan, China, Lot: 20330054610) were used for ELISA analyzes of biomarkers. The intra-assay coefficient of variation (CV), inter-assay CV, and minimum detectable concentrations (MDC) for biomarkers were ≤8%, ≤10%, and 35.7 ng/L for UCHL1, ≤8%, ≤10%, and 0.26 ng/mL for S100B, ≤8%, ≤12% and 5 pg/mL for ADM, <10%, <10% and 1.0 pg/mL for ACTA, ≤8 %, ≤12% and >0.06 ng/mL for NSE, ≤8%, ≤12% and >0.06 ng/mL for GFAP, <8%, <10% and 0.59 ng/mL for CK-B, respectively.

### 2.6. Histopathology

After necropsies and macroscopic examinations of the dead calves, tissue samples taken from different parts of the central nervous system were fixed in 10% buffered formalin for pathological examinations. Then, 5-micron thick sections were taken from the paraffin blocks prepared by routine laboratory methods on a microtome (Reichert-Jung 2030), and all of them were stained with Hematoxylin & Eosin (H&E) [28] and examined under a binocular light microscope (Olympus BX51, Tokyo, Japan). To the description of the severity of lesions, microscopic findings were divided into 4 categories: 0, no lesion; 1, mild; 2, moderate; 3, severe; and 4, very severe.

### 2.7. Immunohistochemistry (IHC)

For immunohistochemical (IHC) staining, 5-micron thick brain sections were stained in Leica Bondmax stainer according to the Bond™ Polymer Refine Detection (Leica Biosystems, Deer Park, IL, USA) kit protocol (Peroxidase Block, Protein Block, Post Primer, Polymer, DAB, Hematoxylin). First, all tissues were dewaxed with heat and dewax solution (Bond™, Leica Biosystems, Deer Park, IL, USA) and rehydrated in serially increasing alcohols (100–70%) (Sigma). After each chemical or marker was used according to the protocol, a special washing solution (Bond™, Leica Biosystems, Deer Park, IL, USA) and/or distilled water was washed 3 times. In order to prevent non-specific staining, peroxidase and protein block application was carried out, and after 30 min of incubation with primary antibody (anti-HIF-1α antibody, Invitrogen, Carlsbad, CA, USA) at room temperature, post primer, and polymer application were performed. All sections were left to react with DAB for 3 min and washed with distilled water. It was then dehydrated by counterstaining with Mayer’s Hematoxylin and sealed with entellan (Merck, Rahway, NJ, USA). The stained tissues were examined under a light microscope (Olympus BX51, Tokyo, Japan), and photographs were taken when deemed necessary (Olympus EP50, Tokyo, Japan). The extent of these reactions was scored as follows: 0: >5% (negative); 1: 6–25% (light); 2: 26–50% (medium); 3: 51–75% (severe); 4: 76–100% (very severe).

### 2.8. Statistical Analysis

SPSS 25 (IBM Corp^®^, 2017, Armonk, NY, USA) statistical program was used to evaluate the data. The Kolmogorov-Smirnov test was used to determine the normality of variables and the homogeneity of variances. Since the variables do not have a normal distribution, the study data are presented as median (min/max). In order to compare calves with perinatal asphyxia and healthy calves, the Wilcoxon test and the Kruskal-Wallis test were performed. The Mann-Whitney U test was used to compare calves with perinatal asphyxia and healthy calves between groups within the same time of the study. Categorical data were analyzed with Chi-Square and Fisher’s Exact tests. The Spearman correlation test was used to determine the correlation between variables. Receiver operating characteristic (ROC) analysis was performed to determine the prognostic cut-off value, sensitivity, and specificity of variables in nonsurvivor and survivor calves with perinatal asphyxia. In addition, the same test was used to evaluate whether brain-related biomarkers have diagnostic significance according to the pathologic results in nonsurvivor calves. Statistical significance was considered as *p* < 0.05, *p* < 0.01, and *p* < 0.001, respectively.

## 3. Results

### 3.1. Clinical Findings

Clinical examination of all calves with perinatal asphyxia showed respiratory distress and tachypnea, weakness, lethargy, cyanotic mucous membranes, tachycardia, hypothermia, and absence of sucking reflex. In addition, severe epistaxis was detected in three calves.

#### Neurological Status

The neurological status score of asphyxiated and healthy calves are presented in Table 2. The neurological status scores of the calves with asphyxia were significantly (*p* < 0.05) lower than the healthy calves at the time of admission and 24th h. In addition, the neurological status scores of calves with asphyxia at the time of admission were significantly (*p* < 0.05) lower compared to the 24, 48, and 72 h.

### 3.2. Blood Gas and CBC Analysis

Arterial blood gas parameters of asphyxiated and healthy calves are presented in Table 3. While the pH, PaO_2_, SO_2_, and BE levels of the calves with asphyxia were significantly (*p* < 0.05) lower at the time of admission compared to the healthy calves, the PaCO_2_, and lactate levels were higher (*p* < 0.05). It was determined that the PaCO_2_ levels of the calves with asphyxia decreased significantly (*p* < 0.05) at the 24th and 48th h compared to the time of admission, while the SO_2_ levels increased significantly (*p* < 0.05) to the 24th h. In addition, it was determined that the pH, BE, and HCO_3_ levels increased significantly (*p* < 0.05), while the lactate concentrations decreased from the 48th h after the treatment in asphyxiated calves. The lactate concentrations of the healthy calves were found to be significantly (*p* < 0.05) higher at the time of admission compared to the 48 and 72 h (Table 3). No statistically significant difference was determined in K, Na, Ca, Cl, Glu, and CBC variables (Supplementary Table S1) between study groups.

### 3.3. Brain-Related Biomarkers Analysis

Biomarker concentrations of asphyxiated and healthy calves are presented in Table 4. S100B and UCHL1 concentrations of calves with asphyxia were significantly higher (*p* < 0.05) than the control group at all time intervals. The ADM concentrations at 24 h, ACTA concentrations at admission, 48 and 72 h, and NSE concentrations at 24, 48, and 72 h, CK-B enzyme activity at admission and 72 h, were significantly (*p* < 0.05) lower in calves with asphyxia compared to healthy calves. There was no statistically significant (*p* > 0.05) difference between the groups in GFAP concentrations.

### 3.4. Correlation Analysis

There was a positive correlation between neurological status score, PaO_2_ (*p* < 0.05), and SO_2_ (*p* < 0.01), and a negative correlation between PaCO_2_, lactate, and ACTA (*p* < 0.01). A positive correlation was found between arterial blood SO_2_ and S100B (*p* < 0.05) (Table 5).

### 3.5. Prognostic Indicators Analysis

#### 3.5.1. Survival Probability

**Figure 1:**
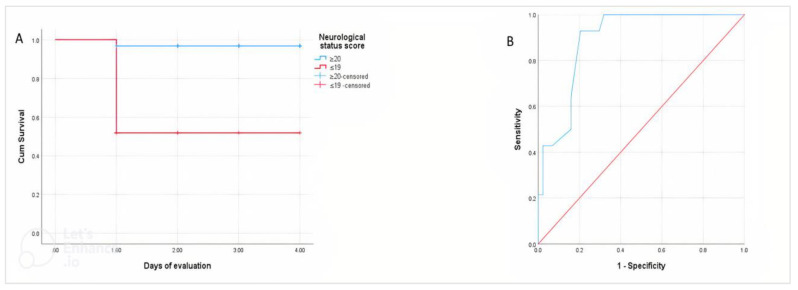
Kaplan-Meier analysis with log-rank test showed that the survival period was significantly shorter in calves with perinatal asphyxia with a neurological status score ≤19 (**A**). Receiver operating characteristic curve (ROC) analysis for the differentiation between the survivor and non-survivor calves with asphyxia based on neurological status score (**B**).

A total of 13 (56%) calves with perinatal asphyxia died during the hospitalization period. Kaplan-Meier analysis showed that the average survival time of asphyxiated calves was 24 h. The cumulative probability of survival calves was 44% for 24, 48, and 72 h. Kaplan-Meier analysis with log-rank test showed that calves with a neurological status score ≤19 had a significantly (*p* < 0.001) shorter survival time than calves with a score ≥20 (Figure 1A).

#### 3.5.2. Neurological Status Score

The ROC analysis findings demonstrated that the neurological status score at the cut-off point of 15, with 90% sensitivity and 80% specificity, has significant (*p* < 0.001) prognosis importance (Figure 1B).

#### 3.5.3. Brain-Related Biomarker

**Figure 2:**
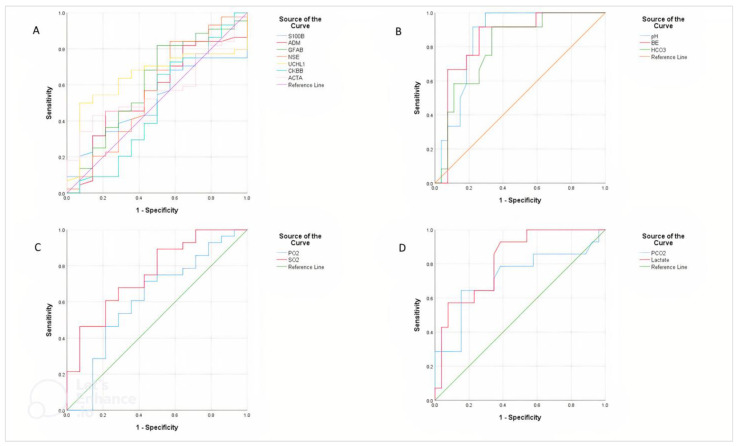
Receiver operating characteristic curve (ROC) analysis for the differentiation between the survivor and non-survivor calves with asphyxia based on the serum brain-related biomarkers (**A**), pH, BE, HCO_3_ (**B**), PaO_2_, SO_2_ (**C**), and PaCO_2_, lactate (**D**) concentrations.

None of UCHL1, S100B, ADM, ACTA, NSE, GFAP, and CK-B were found to be significant (*p* > 0.05) in predicting mortality in calves with asphyxia (Figure 2A).

#### 3.5.4. Blood Gases and Chemistry

The results of the ROC analysis showed that PaCO_2_ (*p* < 0.05) and lactate (*p* < 0.01) were significantly higher in nonsurvivors than survivor’s calves. The pH (*p* < 0.001), HCO_3_ (*p* < 0.01), BE (*p* < 0.01), and SO_2_ (*p* < 0.01) were significantly lower in nonsurvivors than in survivor calves (Table 6, Figure 2B–D)

### 3.6. Pathological Findings

#### 3.6.1. Macroscopic Findings

Macroscopically, non-specific findings such as hyperemia, edema, and dulling in the brain were detected. No cystic structure or macroscopic necrosis foci were found.

#### 3.6.2. Microscopic Findings

**Figure 3:**
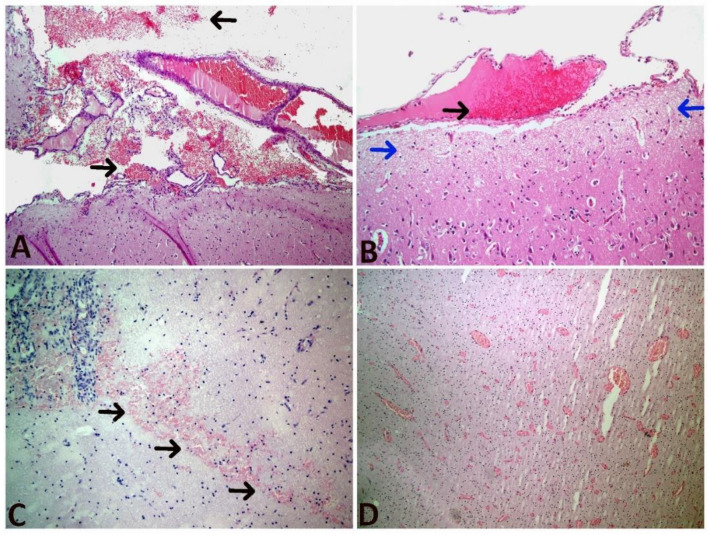
Microscopic photographs (**A**) Edema and hemorrhage in the meninges (arrows), HE, 10×, (**B**) Hyperemia (black arrow) in the meningeal veins and edema in the submeningeal region (blue arrows), HE, 10×, (**C**) Hemorrhage spreading to the neuropil tissue (arrows), HE, 20×, (**D**) Severe hyperemia and vasodilation, HE, 10×.

**Figure 4:**
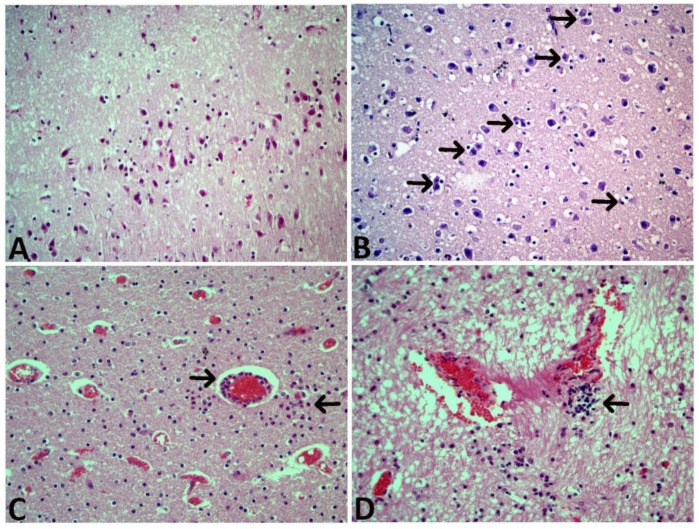
Microscopic photographs (**A**) Ischemic neuronal changes and neuronophagia, HE, 20×, (**B**) Neuronophagia (black arrow), HE, 40×, (**C**) Perivascular neutrophil and mononuclear cell infiltration (arrows), HE, 40×, (**D**) Cavitation area and local Mononuclear cell infiltration with gliosis (arrow), HE, 40×.

The microscopic examination findings are presented in Figure 3 and Figure 4. Ischemic neuronal changes (IND, increased eosinophilia/degeneration, and necrosis); neuronophagia (NF); gliosis, mononuclear cell infiltration (MND); Scoring for hyperemia, endothelial cell swelling (ECS), edema and bleeding were the most frequently observed conditions. These findings confirmed developed hypoxic ischemic encephalopathy in calves with perinatal asphyxia (Supplementary Table S2).

#### 3.6.3. Immunohistochemical

**Figure 5:**
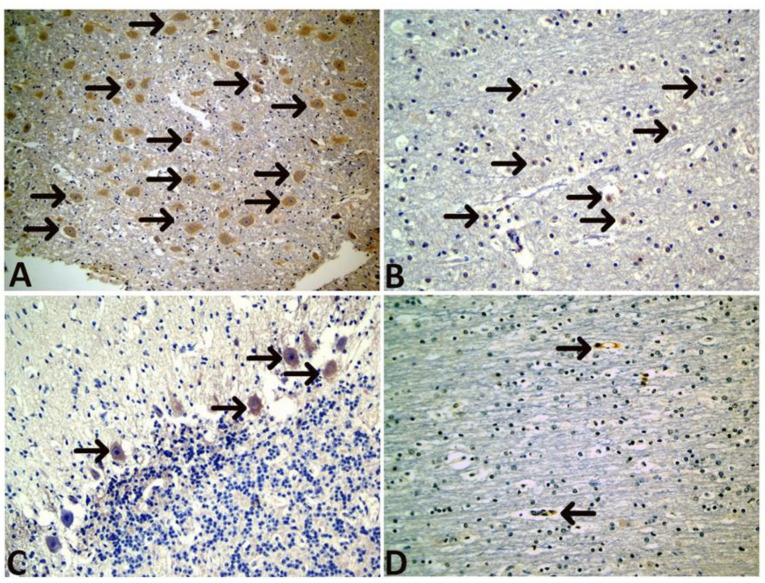
Immunohistochemical findings. (**A**) Immune positive reaction in neurons (arrows), HIF-1α, 20×, (**B**) Immune positive reaction in glia cells (arrows), HIF-1α, 20×, (**C**) Immune positive reaction in Purkinje cells (arrows), HIF-1α, 40×, (**D**) Vascular Immunopositivity in endothelial cells and their walls, (arrows), HIF-1α, 20×.

The immunohistochemical examination findings are presented in Figure 5. In the immunohistochemical staining performed with the primary antibody of hypoxia-inducible factor 1 alpha (HIF-1α), immunopositivity was determined in endothelial cells (nuclear), glia cells (cytoplasmic) and neurons (nuclear and cytoplasmic). No positive staining was observed in the negative control slides (Supplementary Table S3).

## 4. Discussion

In the present study, the concentrations of serum brain damage biomarkers were evaluated in both healthy and asphyxiated perinatal calves for the first time. By using histological and immunohistochemical methods, we determined that hypoxic-ischemic encephalopathy developed in non-survived calves with perinatal asphyxia.

Asphyxia is a life-threatening condition characterized by hypoxemia due to respiratory dysfunction because of prolonged parturition or aspiration of the amniotic fluid aspiration [29]. In the absence of oxygen, primary and secondary energy disorders occur in the neurons so that the brain cells cannot be nourished and die [5]. As a consequence of the decrease in blood flow to the brain due to primary energy disorder, the level of oxygen and glucose entering the brain tissue decreases [4]. In this situation, a lack of energy and an increase in lactate production in the brain tissue led to the development of hypoxic-ischemic encephalopathy [30].

Clinically, in newborn foals with hypoxic-ischemic encephalopathy, tremor, excitability, fatigue, insomnia, lethargy, clonic seizures, random wandering, abnormal vocalization, loss of suckling, dysphagia, blindness, unconsciousness, nystagmus, eye deviation, head tilting, irregular breathing, respiratory distress, spastic dysmetric gait, coma, and death have been reported [31]. Asphyxiated calves showed symptoms of weakness, lethargy, cyanotic mucous membranes, tachycardia, hypothermia, a weak or absent suckling reflex, blindness, convulsions, loss of consciousness, and death [3,25,32]. In the present study, calves with perinatal asphyxia showed severe respiratory distress, reduced body temperature, increased respiratory and heart rate, decrease in muscular tone, lateral recumbency, decrease or absence of sucking reflex, decrease or absence of pupillary and corneal reflex, loss of consciousness, convulsions, or clinical symptoms of mental depression and coma. Moreover, three calves developed severe epistaxis. The clinical findings we observed in calves with perinatal asphyxia were compatible with the previous studies [3,25,31,32].

In human medicine, hypoxic-ischemic encephalopathy due to asphyxia is responsible for 10–60% of perinatal mortality [6,33]. Additionally, it has been reported that in newborn calves, respiratory and metabolic acidosis due to asphyxia is the main cause of perinatal mortality [3,34,35]. In the present study, 12 (44%) of 25 calves with perinatal asphyxia survived, whereas 13 of them (56%) nonsurvived. One of the most important causes of death in calves with perinatal asphyxia is hypoxic-ischemic encephalopathy due to severe hypoxia and respiratory dysfunction [1,36,37]. It was observed that there was no improvement in the clinical picture of 13 non survived calves with perinatal asphyxia. When the neurological status score and mortality rate of calves with perinatal asphyxia were evaluated together, at the time of admission and 24th h calculated scores of the calves in the asphyxia group were significantly lower than the calves in the control group. There were 13 calves with poor neurological status scores that did not survive during the first 24 h of hospitalization. In our opinion, the high sensitivity and specificity of the neurological status score (90% and 80%, respectively) in predicting mortality in calves with asphyxia make it an effective tool in the clinical setting. It might be concluded that calves with a score of less than 15 have a significantly high mortality rate.

The α and β subunits of hypoxia-inducible factor 1 (HIF-1) form an active heterodimer under hypoxic circumstances. Histopathological examinations and immunohistochemical detection of HIF-1α were reported to be useful in demonstrating hypoxic tissue damage in the brain [38,39,40]. Immunohistochemical findings were found to be interesting and promising in the post-mortem diagnosis of acute cerebral hypoxia and ischemia [41]. Histopathologically, it was determined that more than 20% of infants with perinatal asphyxia developed lethal hypoxic-ischemic encephalopathy, and more than 25% developed permanent nervous system disorder [10]. Some previous research in perinatal calves [34,36] detected asphyxic pathological changes in the brain tissue in 73–75% of cases. In parallel, Schuijt [42] found histopathological changes in 58.3% of calves that died during the perinatal period. In the present study, histopathological examination of the brain of 13 calves with perinatal asphyxia showed signs of mild to severe hypoxic-ischemic encephalopathy such as ischemic neuronal changes (increased eosinophilia/degeneration and necrosis), neuronophagia, gliosis, mononuclear cell infiltration, hyperemia, endothelial cell swelling, edema, and hemorrhage. Also, immunohistochemical staining was performed directly on brain tissue with HIF-1α primary antibody, and immuno-positivity was observed in endothelial cells (nuclear), glia cells (cytoplasmic), and neurons (nuclear and cytoplasmic). Against low oxygen levels, the expression of HIF-1α at various levels in neurons is indicative of exposure to hypoxia. Histopathological and immunohistochemical examination results show that hypoxic-ischemic encephalopathy develops in calves with perinatal asphyxia. From this point of view, we think that these two examination methods are reliable diagnostic methods that complement and support each other in the determination of hypoxic damage in the brain.

Mixed acidosis (respiratory-metabolic acidosis) with hypoxia and hypercapnia is a common finding in calves with perinatal asphyxia [1,2,3,25,27,43]. In the present study, at the time of admission, pH, PaO_2_, SO_2_, and BE levels of calves with perinatal asphyxia were significantly lower than the control group, and the PaCO_2_ and lactate were found to be higher. In addition, the pH, SO_2_, HCO_3_, and BE levels were found to be significantly lower, and PaCO_2_ and lactate were found to be higher in the nonsurvivors compared to the survivors. On the other hand, a positive correlation was established between the neurological status score, PaO_2_ and SO_2_, and a negative correlation between PaCO_2_ and lactate. When taking these findings into account, it can be stated that respiratory-metabolic acidosis develops in calves with perinatal asphyxia and hypoxemia affects the neurological status and consciousness level of calves. In addition, postnatal acidosis and hypoxia in calves with asphyxia can be considered important indicators of survival [2,25,27,35]. A recent study in calves with asphyxia performed by İder et al. [3] can confirm our results.

In recent years, some brain damage-related biomarkers have been used to detect hypoxic-ischemic brain damage in asphyxiated infants [8,10,44,45]. In this regard, in veterinary medicine, some biomarkers (UCHL1, S100B, NSE) have been evaluated only in newborn foals [12] and pigs [46] for the diagnosis of brain damage [9]. Since no studies were found in calves, the discussion of our results was made by human literature.

Ubiquitin carboxy-terminal hydrolase 1 (UCHL1) is a soluble brain protein with ligase and hydrolase multiple activities expressed in the central nervous system and neuroendocrine cells [47]. It has been reported that UCHL1 is a useful marker that can be helpful in diagnosing acute brain injury and determining the severity of the damage in infants with hypoxic-ischemic encephalopathy [48]. UCHL1 and GFAP have been found to have neuroprognostic importance in infants with neonatal encephalopathy [49]. On the other hand, it was stated that serum UCHL1 concentration increased significantly in foals with neonatal hypoxic-ischemic encephalopathy compared to healthy foals, and UCHL1 could be an important diagnostic indicator in detecting brain damage [12]. Douglas-Escobar et al. [9] reported that the UCHL1 is a reliable biomarker for detecting brain damage, and its specificity for the diagnosis of neonatal hypoxic-ischemic encephalopathy is 100%. In the present study, a statistically significant increase was determined in the UCHL1 concentrations of calves with perinatal asphyxia compared to healthy calves at the time of admission, 24, 48, and 72 h. After treatment conduction, serum UCHL1 concentrations of calves with perinatal asphyxia gradually decreased at 24, 48, and 72 h compared to the time of admission. A significant increase in UCHL1 concentrations in asphyxiated calves may be an indicator of hypoxic-ischemic damage [9,12,48,49], and we believe that it can be a useful diagnostic marker in the detection of hypoxic-ischemic encephalopathy due to asphyxia in perinatal calves.

S100B measurement in blood and cerebrospinal fluid is considered reliable in the evaluation of developing brain damage in perinatal infants with asphyxia [14]. In term and preterm infants with hypoxic-ischemic encephalopathy, S100B concentration was found to be elevated within the first 72 h [50]. In contrast, Nagdyman et al. [51] reported that S100B is rapidly released in hypoxic brain injury and returns to normal ranges within 48 h. Previous studies showed that in the umbilical cord blood of infants born with neonatal asphyxia S100B and lactate concentrations were increased, and these markers could be helpful as an early predictive marker for diagnosis of neonatal hypoxic-ischemic encephalopathy [52,53]. In the present study, a statistically significant increase was found in the S100B concentration of calves with perinatal asphyxia compared to healthy calves at the time of admission, 24, 48, and 72 h. Higher S100B concentrations in calves with perinatal asphyxia compared to healthy calves have been associated with the development of hypoxic-ischemic encephalopathy [50,51]. In our study, the concomitant elevation of S100B, lactate [52,53], and UCHL1 concentrations in calves with perinatal asphyxia supports the development of hypoxic-ischemic encephalopathy.

ADM plays a role as a regulator to promote neural regeneration in neural damage [54]. It has been reported that plasma ADM concentrations increase in patients with acute ischemic stroke, and this increase continues for a long time [55,56]. It has been stated that the increase in ADM concentrations may vary according to the severity of the neural damage and the extent of the cerebrovascular infarction [57]. On the other hand, Mitome-Mishima et al. [58] reported that ischemic white matter damage, which develops as a result of prolonged cerebral hypoperfusion in mice, causes excessive oxidative stress and increased free radicals, resulting in the insufficient release of ADM. In the present study, contrary to previous studies [55,56,57], a statistically significant decrease was observed in the ADM concentration in calves with perinatal asphyxia at the 24th h compared to healthy calves. We believe that the decrease in serum ADM concentrations of asphyxiated perinatal calves may be associated with hypoxia and the release of intense oxidative stress products [59]. Especially in newborns, oxidative stress products reduce high oxygen consumption and low antioxidant levels during the transition from the fetal to neonatal period, the insufficient ability of the brain to remove free radicals and increased sensitivity to them cause damage to the central nervous system tissue [2,60,61]. In our opinion, the excessive production of free radicals in calves with perinatal asphyxia not only causes neuronal damage but also leads to the slowdown of neuron development and reduces the release of these markers (ADM, ACTA, CK-B) into the bloodstream.

ACTA concentrations were found to be increased in the blood, urine, and cerebrospinal fluid of infants that developed hypoxic ischemic encephalopathy due to neonatal asphyxia [62]. In addition, increased ACTA concentrations in umbilical cord blood have been found in infants with mild or moderate neonatal hypoxic-ischemic encephalopathy and poor nervous system development [63]. In the present study, serum ACTA concentrations were significantly lower at the time of admission, 48, and 72 h in calves with perinatal asphyxia. Contrary to previous studies [62,63], the detected low ACTA concentrations in our study may be originated from the studied species (calf), the destruction of this protein by the over-released oxidative stress products as stated in ADM [58], the overuse of ACTA during nervous tissue recovery [16,64], and immature brain structure in newborns [46].

NSE concentrations were found to be significantly increased in infants with neonatal asphyxia [17]. It has been reported that serum and cerebrospinal fluid concentrations of NSE can be used to predict the prognosis and to determine the extent of neuronal damage in infants with hypoxic-ischemic encephalopathy [18,19]. In the present study, serum NSE concentrations were significantly lower (*p* < 0.05) at 24, 48, and 72 h in calves with perinatal asphyxia. Contrary to studies in newborn infants with hypoxic-ischemic encephalopathy [17,18,19], similar to our observation, serum NSE was found to be low in newborn pigs with asphyxia [46]. The authors concluded that because the pigs were immature, the neonatal brain contained less glial, axonal mass, and myelinization. The previous findings in newborn pigs with asphyxia and the description of the immature brain structure may contribute to explaining the lower serum NSE concentrations in calves with perinatal asphyxia.

Glial fibrillary acidic protein (GFAP) is a monomeric filament protein found in astroglial cells. High serum GFAP concentrations have been reported in infants with hypoxic-ischemic encephalopathy [49,65]. In contrast, no difference was found between umbilical cord blood GFAP concentrations of moderate HIE (stage II), severe HIE (stage III), and healthy infants, and also no correlation was found between GFAP concentrations and HIE severity [66]. In the present study, there was no statistically significant difference in GFAP concentrations of calves with perinatal asphyxia compared to healthy calves at set intervals. In previous studies, it has been stated that even if structural brain lesions develop in newborns, GFAP concentrations may not increase [20,66]. Despite the development of hypoxic-ischemic encephalopathy in calves with perinatal asphyxia, the lack of expected increase in serum GFAP concentrations may be related to the fact that GFAP is usually found in astrocytes and their structure is not broken down unless severe damage occurs [67], and therefore, GFAP is not released sufficiently into the bloodstream.

Creatine kinase-B is an isoenzyme found in neurons and astrocytes and used together with S100B in the diagnosis of brain damage in the neonatal period [51]. It was determined that CK-B enzyme activity increased significantly in infants with perinatal asphyxia that developed neurological disorders [21,68]. In another study, it was determined that CK-B enzyme activity increased for 1 to 3 days and decreased rapidly in infants that died because of severe brain damage [69]. In the present study, CK-B enzyme activity in calves with perinatal asphyxia was significantly lower at the time of admission and 72 h compared to healthy calves. Lower CK-B enzyme activity in calves with perinatal asphyxia may be due to oxidative stress products that intensely produce during hypoxemic episodes, destroy this enzyme, and decrease its activity [58]. This situation may explain why CK-B enzyme activity increases in a short time and then decreases rapidly [69].

## 5. Conclusions

Histopathologically, it was confirmed that hypoxic-ischemic encephalopathy developed in calves with perinatal asphyxia. The mortality risk in calves with perinatal asphyxia with a neurologic status score < 15 was found to be high. Some arterial blood gas and chemistry variables were useful indicators of mortality prediction in calves with perinatal asphyxia. Most important, UCHL1 and S100B concentrations were found to be useful markers for the determination of hypoxic-ischemic encephalopathy in calves with perinatal asphyxia. In contrast to our expectation, serum ADMA, ACTA, NSE, GFAP, and CK-B concentrations were found to be low. It may be related to excessive oxidative stress and severe damage to the brain of newborns due to high oxygen consumption and low antioxidant levels during the transition from the fetal to the neonatal period.

## Figures and Tables

**Table 1:** Scoring variables and ranges of the neurological status evaluation in perinatal calves.

Score	3	2	1
Position	Normal gait	Sternal position	Lateral recumbency
Mental status	Alertness and responsive to environment	Depression	Comatose
Pupillary light reflex (PLR)	Normal PLR	Slow PLR	Unresponsive mydriasis
Corneal reflex (CR)	Normal CR	Slow CR	Unresponsive
Responsive to auditory stimuli	Responsive to auditory stimuli	Decrease responsive to auditory stimuli	Unresponsive
Level of consciousness	Responsive	Delirium	Unresponsive
Sucking reflex	Strong	Weak	Absence
Tonus of muscles	Normal	Hypotonia	Atonia

**Table 2:** Neurological status score in healthy and calves with perinatal asphyxia.

Variable		Time of Evaluation (Hours)			
		Admission (n_H_: 10, n_A_: 25)	24 (n_H_: 10, n_A_: 12)	48 (n_H_: 10, n_A_: 12)	72 (n_H_: 10, n_A_: 12)
Score	Healthy	24	24	24	24
	Asphyxia	12.00 ^a^ (8–20)	22.00 ^b^ (15–24)	24.00 ^b^ (16–24)	24.00 ^b^ (22–24)
	*p*-Value	0.000	0.001		

n_H_: number of calves included in the healthy group, n_A_: number of calves included in the asphyxia group. Different letters (^a,b^) in the same line are statistically significant (*p* < 0.05).

**Table 3:** Arterial blood gas and chemistry variables of healthy and calves with perinatal asphyxia.

Variable	Time of Evaluation (Hours)				
		Admission (n_H_: 10, n_A_: 25)	24 (n_H_: 10, n_A_: 12)	48 (n_H_: 10, n_A_: 12)	72 (n_H_: 10, n_A_: 12)
pH	Healthy		7.43 (7.40–7.57)	7.43 (7.35–7.50)	7.46 (7.39–7.70)
	Asphyxia	7.21 (6.75–7.41) ^a^	7.44 (7.41–7.45) ^ab^	7.46 (7.35–7.50) ^b^	7.39 (7.30–7.45) ^ab^
	*p*-Value	0.000			
PaCO_2_ (mmHg)	Healthy	41.10 (25.10–48.50)	37.45 (27.40–46.30)	37.40 (34.30–46.80)	33.40 (17.20–46.40)
	Asphyxia	55.50 (35.80–89.10) ^a^	36.80 (27.90–40.00) ^b^	40.40 (30.10–55.40) ^b^	40.30 (38.30–51.20) ^ab^
	*p*-Value	0.000			
PaO_2_ (mmHg)	Healthy	49.20 (23.40–97.10)	52.05 (23.10–68.50)	49.05 (25.20–80.40)	61.20 (28.70–150)
	Asphyxia	32.80 (18.60–69.60)	60.30 (57.70–62.90)	39.80 (23.70–65.60)	34.50 (20.70–57.10)
	*p*-Value	0.008			
SO_2_ (%)	Healthy	96.45 (58.90–101.30)	96.20 (56.20–98.80)	94.70 (56.80–99.90)	95.85 (65.60–101.50)
	Asphyxia	77.10 (4.10–97.40) ^a^	98.40 (98.30–99.20) ^b^	91.70 (34.30–100.00) ^ab^	85.20 (44.80–96.40) ^ab^
	*p*-Value	0.000			
K (mmol/L)	Healthy	4.35 (3.90–4.80)	4.50 (3.90–4.5)	4.30 (3.90–5.00)	4.30 (2.90–5.50)
	Asphyxia	4.05 (2.60–6.13)	4.00 (3.40–4.00)	4.00 (2.60–4.50)	4.00 (3.90–4.10)
	*p*-Value				
Na (mmol/L)	Healthy	146.50 (140–153)	144.50 (141–157)	146.00 (141–152)	149.50 (141–167)
	Asphyxia	147.00 (136–159)	149.00 (145–161)	147.00 (139–164)	149 (149–151)
	*p*-Value				
Ca (mmol/L)	Healthy	1.03 (0.68–1.28)	0.90 (0.46–1.17)	0.90 (0.63–1.30)	0.75 (0.55–1.17)
	Asphyxia	1.00 (0.59–1.44)	1.10 (1.03–1.17)	0.94 (0.51–1.15)	0.85 (0.74–1.17)
	*p*-Value				
Cl (mmol/L)	Healthy	103.00 (100–115)	104.00 (99–107)	102.50 (98–106)	106.50 (99–114)
	Asphyxia	103.00 (87–110)	104.00 (100–112)	102.00 (91–111)	108.00 (100–111)
	*p*-Value				
Glu (mg/dL)	Healthy	103.00 (54–137)	111.00 (91–138)	108.00 (76–123)	107.00 (78–139)
	Asphyxia	75.00 (0–264)	76.00 (65–121)	105.00 (72–138)	66.00 (56–115)
	*p*-Value				
Lac (mmol/L)	Healthy	4.00 (2.60–5.20) ^a^	3.30 (1.80–4.00) ^ab^	2.05 (1.10–4.60) ^b^	1.85 (0.90–2.90) ^b^
	Asphyxia	7.90 (1.60–29.00) ^a^	9.10 (7.50–10.70) ^ab^	2.10 (1.20–5.00) ^b^	3.00 (0.70–7.00) ^ab^
	*p*-Value	0.001			
BE (mmol/L)	Healthy	0.25 (−7.20–5.40)	1.25 (−1.40–6.20)	1.10 (−1.20–6.20)	1.15 (−6.40–6.80)
	Asphyxia	−5.60 (−18.00–1.80) ^a^	−1.70 (−4.90–3.50) ^ab^	4.70 (−3.70–14.80) ^b^	−1.50 (−3.90–1.40) ^ab^
	*p*-Value	0.002			
HCO_3_ (mmol/L)	Healthy	25.25 (17–30)	25.20 (22.60–30.50)	25.60 (23.20–30.40)	24.55 (17.60–31.00)
	Asphyxia	23.40 (14.00–27.90) ^a^	23.00 (19.10–27.60) ^ab^	28.00 (20.90–38.00) ^b^	25.00 (23.40–27.90) ^ab^
	*p*-Value				

n_H_: number of calves included in the healthy group, n_A_: number of calves included in the asphyxia group, PaCO_2_ (partial arterial carbon dioxide pressure), PaO_2_ (partial arterial oxygen pressure), SO_2_ (oxygen saturation), K (potassium), Na (sodium), Ca (calcium), Cl (chlorine), Glu (glucose), Lac (lactate), BE (base deficit), HCO_3_ (bicarbonate); Different letters (^a,b^) in the same line are statistically significant (*p* < 0.05).

**Table 4:** Biomarker concentrations result in healthy and calves with perinatal asphyxia.

Variable	Time of Evaluation (Hours)				
		0.saat (n_H_: 10, n_A_: 25)	24.saat (n_H_: 10, n_A_: 12)	48.saat (n_H_: 10, n_A_: 12)	72.saat (n_H_: 10, n_A_: 12)
UCHL1 (ng/L)	Healthy	815.25 (504.94–1066.98) ^a^	784.71 (414.73–1103.11) ^ab^	703.77 (454.14–918.44) ^ab^	558.81 (33.13–922.01) ^b^
	Asphyxia	1918.42 (1267.64–3092.14)	1679.87 (1296.22–6336.06)	1428.56 (1261.93–4400.46)	1751.06 (1199.29–5237.30)
	*p*-Value	0.000	0.000	0.000	0.000
S100B (ng/mL)	Healthy	12.99 (8.33–20.97) ^a^	9.16 (6.47–13.71) ^b^	9.85 (6.37–11.29) ^ab^	8.76 (5.79–13.80) ^b^
	Asphyxia	30.34 (19.76–46.25)	30.24 (24.79–106.76)	26.56 (21.59–88.16)	63.99 (23.82–89.13)
	*p*-Value	0.000	0.000	0.000	0.000
ADM (pg/mL)	Healthy	156.89 (92.21–305.80)	200.51 (53.59–320.89)	204.85 (69.32–378.92)	202.27 (124.67–317.82)
	Asphyxia	144.96 (19.51–287.69)	128.01 (100.49–183.49)	152.45 (28.91–534.59)	147.80 (107.97–247.20)
	*p*-Value		0.010		
ACTA (pg/mL)	Healthy	6713.74 (4177.42–7207.84) ^a^	5531.27 (3479.30–7493.42) ^ab^	4640.49 (2393.00–6395.36) ^ab^	4314.41 (2599.97–5721.20) ^b^
	Asphyxia	4256.21 (2087.61–8596.39) ^a^	5068.65 (3946.66–7062.84) ^a^	2269.94 (331.93–5536.58) ^b^	2565.06 (1211.90–3577.87) ^b^
	*p*-Value	0.031		0.008	0.001
NSE (ng/mL)	Healthy	4.10 (2.14–8.77)	5.69 (2.06–9.88)	5.05 (2.30–10.05)	5.01 (3.38–8.95)
	Asphyxia	3.48 (0.90–5.49)	2.94 (2.07–4.14)	3.50 (0.83–6.30)	3.67 (2.42–4.54)
	*p*-Value		0.020	0.024	0.002
GFAP (ng/mL)	Healthy	3.57 (0.39–7.23)	3.62 (0.38–7.37)	3.96 (0.91–6.60)	2.98 (2.36–5.84)
	Asphyxia	3.54 (0.34–6.22)	2.74 (1.84–4.04)	3.68 (0.52–9.08)	4.16 (2.10–5.38)
	*p*-Value				
CK-B (ng/mL)	Healthy	7.14 (4.21–9.68)	8.05 (4.00–11.85)	7.09 (1.81–11.46)	8.08 (3.70–12.18)
	Asphyxia	3.39 (0.40–17.69)	5.44 (2.93–17.10)	4.90 (2.41–13.49)	2.77 (0.51–13.49)
	*p*-Value	0.021			0.024

n_H_: number of calves included in the healthy group, n_A_: number of calves included in the asphyxia group, S100B (calcium-binding protein B), ADM (adrenomodullin), GFAP (glial fibrillary acidic protein), NSE (neuron-specific enolase), UCHL1 (ubiquitin carboxy-terminal hydrolysis 1), CK-B (creatine kinase-brain), ACTA (activitin A); Different letters (^a,b^) in the same line are statistically significant (*p* < 0.05).

**Table 5:** Correlations between arterial blood gas variables, brain-related biomarkers, and neurological status score in healthy and calves with perinatal asphyxia.

Variable	UCHL1	S100B	ADM	ACTA	NSE	GFAP	CK-B	Neurological Status Score
PaCO_2_	0.117	0.031	0.178	0.220	0.096	0.113	0.147	−0.657 **
PaO_2_	0.028	0.278	−0.201	0.042	−0.013	−0.238	−0.188	0.328 *
SO_2_	0.001	0.323 *	−0.230	0.020	−0.138	−0.271	−0.115	0.507 **
Lactate	0.235	0.155	0.159	0.143	0.196	0.165	0.139	−0.626 **
Neurological status score	−0.161	0.068	−0.004	−0.497 **	0.051	0.007	0.063	1.00

* *p* < 0.05, ** *p* < 0.01.

**Table 6:** The area under the curve (AUC), standard error, confidence interval (95%), optimum cut-off values, respective sensitivity, and specificity of mortality prediction in nonsurvivor calves.

Variable	AUC	Standard Error	*p*-Value	Asymptotic 95% Confidence Interval		Sensitivity	Specificity	Cut-Off Value
				Lower Band	Upper Bound			
PaO_2_	0.617	0.098	0.220	0.426	0.809	60	65	35.60
SO_2_	0.755	0.079	0.008	0.601	0.909	67	72	78.75
PaCO_2_	0.720	0.093	0.023	0.537	0.902	71	66	50
Lactate	0.820	0.067	0.001	0.690	0.951	85	66	6.4
pH	0.853	0.060	0.000	0.735	0.971	91	78	7.29
HCO_3_	0.801	0.073	0.003	0.659	0.943	83	67	24.15
BE	0.843	0.067	0.001	0.711	0.974	91	75	−2.45

## Data Availability

Not applicable.

## Supplementary Materials

The following supporting information can be downloaded at: https://www.mdpi.com/article/10.3390/ani12223223/s1, Table S1: Hemogram variables of healthy and calves with perinatal asphyxia; Table S2: Scores of histopathological lesions in nonsurvivor calves with perinatal asphyxia; Table S3: Immunohistochemistry scores in nonsurvivor calves with perinatal asphyxia.

## References

[1] Bleul U. (2009). Respiratory distress syndrome in calves. Vet. Clin. N. Am. Food Anim..

[2] Ok M., Yıldız R., Traş B., Başpınar N., Akar A. (2021). Oxidative stress and acute phase response status during treatment in premature calves with respiratory distres syndrome. J. Hell. Vet. Med. Soc..

[3] Ider M., Naseri A., Ok M., Gulersoy E., Bas T.M., Uney K., Parlak T.M., Abdelaziz A. (2022). Serum sRAGE and sE-selectin levels are useful biomarkers of lung injury and prediction of mortality in calves with perinatal asphyxia. Theriogenology.

[4] Shalak L., Perlman J.M. (2004). Hypoxic-ischemic brain injury in the term infant-current concepts. Early Hum. Dev..

[5] Cotten C.M., Shankaran S. (2010). Hypothermia for hypoxic-ischemic encephalopathy. Expert Rev. Obstet. Gynecol..

[6] Badr Zahr L.K., Purdy I. (2006). Brain injury in the infant: The old, the new, and the uncertain. J. Perinat. Neonatal Nurs..

[7] Gazzolo D., Abella R., Marinoni E., Di Iorio R., Li Volti G., Galvano F., Sabatini M. (2009). New markers of neonatal neurology. J. Matern-Fetal Neonatal Med..

[8] Bennet L., Booth L., Gunn A.J. (2010). Potential biomarkers for hypoxic–ischemic encephalopathy. Semin. Fetal Neonatal Med..

[9] Douglas-Escobar M.V., Weiss M.D. (2013). Biomarkers of brain injury in the premature infant. Front. Neurol..

[10] Bersani I., Auriti C., Ronchetti M.P., Prencipe G., Gazzolo D., Dotta A. (2015). Use of early biomarkers in neonatal brain damage and sepsis: State of the art and future perspectives. BioMed Res. Int..

[11] Siman R., Toraskar N., Dang A., McNeil E., McGarvey M., Plaum J., Maloney E., Grady M.S. (2009). A panel of neuron-enriched proteins as markers for traumatic brain injury in humans. J. Neurotrauma.

[12] Ringger N.C., Giguere S., Morresey P.R., Yang C., Shaw G. (2011). Biomarkers of brain injury in foals with hypoxic-ischemic encephalopathy. J. Vet. Intern. Med..

[13] Rickmann M., Wolff J.R. (1995). S100 protein expression in subpopulations of neurons of rat brain. Neuroscience.

[14] Blennow M., Sävman K., Ilves P., Thoresen M., Rosengren L. (2001). Brain-specific proteins in the cerebrospinal fluid of severely asphyxiated newborn infants. Acta Paediatr..

[15] Di Iorio R., Marinoni E., Lituania M., Serra G., Letizia C., Cosmi E.V., Gazzolo D. (2004). Adrenomedullin increases in term asphyxiated newborns developing intraventricular hemorrhage. Clin. Biochem..

[16] Mukerji S.S., Katsman E.A., Wilber C., Haner N.A., Selman W.R., Hall A.K. (2007). Activin is a neuronal survival factor that is rapidly increased after transient cerebral ischemia and hypoxia in mice. J. Cereb. Blood Flow Metab..

[17] Massaro A.N., Chang T., Kadom N., Tsuchida T., Scafidi J., Glass P., McCarter R., Baumgart S., Vezina G., Nelson K.B. (2012). Biomarkers of brain injury in neonatal encephalopathy treated with hypothermia. J. Pediatr..

[18] Celtik C., Acunbaş B., Öner N., Pala O. (2004). Neuron-specific enolase as marker of the severity and outcomeof hypoxic ischemic encephalopathy. Brain Dev..

[19] Lv H., Wang Q., Wu S., Yang L., Ren P., Yang Y., Gao J., Li L. (2015). Neonatal hypoxic ischemic encephalopathy-related biomarkers in serum and cerebrospinal fluid. Clin. Chim. Acta.

[20] Ennen C.S., Huisman T.A., Savage W.J., Northington F.J., Jennings J.M., Everett A.D., Graham E.M. (2011). Glial fibrillary acidic protein as a biomarker for neonatal hypoxicischemic encephalopathy treated with whole-body cooling. Am. J. Obstet. Gynecol..

[21] Sweet D.G., Bell A.H., McClure G., Wallace I.J., Shields M.D. (1999). Comparison between creatine kinase brain isoenzyme (CKBB) activity and Sarnat score for prediction of adverse outcome following perinatal asphyxia. J. Perinat. Med..

[22] Mohri M., Sharifi K., Eidi S. (2007). Hematology and serum biochemistry of Holstein dairy calves: Age related changes and comparison with blood composition in adults. Res. Vet. Sci..

[23] Probo M., Giordano A., Moretti P., Opsomer G., Fiems L.O., Veronesi M.C. (2012). Mode of delivery is associated with different hematological profiles in the newborn calf. Theriogenology.

[24] Dillane P., Krump L., Kennedy A., Sayers R.G., Sayers G.P. (2018). Establishing blood gas ranges in healthy bovine neonates differentiated by age, sex, and breed type. J. Dairy Sci..

[25] Yildiz R., Ok M. (2017). Clinical efficacy of combinations of nebulised fluticasone, salbutamol and furosemide on lung function in premature calves with respiratory distress syndrome. Vet. Med..

[26] Ider M., Naseri A., Ok M., Uney K., Erturk A., Durgut M.K., Parlak T.M., Ismaıloglu N., Kapar M.M. (2021). Biomarkers in premature calves with and without respiratory distress syndrome. J. Vet. Intern. Med..

[27] Ok M., Yıldız R., Traş B., Başpınar N., Akar A. (2020). Effect of nebulized formeterol, ipratropium bromid, and furosemid in combination combination with fluticasone propionate on arterial blood gases prematüre calves with respiratory distres syndrome. J. Hell. Vet. Med. Soc..

[28] Luna L.G. (1968). Manual of Histologic Staining Methods of the Armed Forces Institute of Pathology.

[29] Bleul U., Lejeune B., Schwantag S., Kähn W. (2007). Blood gas and acid-base analysis of arterial blood in 57 newborn calves. Vet. Rec..

[30] Hanrahan J.D., Sargentoni J., Azzopardi D. (1996). Cerebral metabolism within 18 hours of birthasphyxia: A proton magnetic resonance spectroscopy study. Pediatr. Res..

[31] Palmer C., Roberts R.L., Young P.I. (2004). Timing of neutrophil depletion influences long-term neuroprotection in neonatal rat hypoxic-ischemic brain injury. Pediatr. Res..

[32] Bleul U.T., Bircher B.M., Kahn W.K. (2008). Effect of intranasal oxygen administration on blood gas variables and outcome in neonatal calves with respiratory distress syndrome: 20 cases (2004–2006). J. Am. Vet. Med. Assoc..

[33] Namusoke H., Nannyonga M.M., Ssebunya R., Nakibuuka V.K., Mworozi E. (2018). Incidence and short term outcomes of neonates with hypoxic ischemic encephalopathy in a Peri Urban teaching hospital, Uganda: A prospective cohort study. Matern. Health Neonatol. Perinatol..

[34] Balikçi E., Yıldız A. (2009). Effects on arterial blood gases and some clinical parameters of caffeine, atropine sulphate or doxapram hydrochloride in calves with neonatal asphyxia. Rev. Med. Vet..

[35] Bleul U., Götz E. (2013). The effect of lactic acidosis on the generation and compensation of mixed respiratory-metabolic acidosis in neonatal calves. Vet. Rec..

[36] Szenci O. (2003). Role of acid-base disturbances in perinatal mortality of calves: Review. Vet. Bull..

[37] Murray P.G., Stewart M.J. (2008). Use of nasal continuous positive airway pressure during retrieval of neonates with acute respiratory distress. Pediatrics.

[38] Bergeron M., Yu A.Y., Semenza G.L., Ferrireo D.M., Sharp F.R. (2000). Role of hypoxia-inducible factor-1 in hypoxia-induced ischemic tolerance in neonatal rat brain. Ann. Neurol..

[39] Bergeron M., Yu A.Y., Solway K.E., Semenza G.L., Sharp F.R. (1999). Induction of hypoxia-inducible factor-1 (HIF-1) and its target genes following focal ischaemia in rat brain. Eur. J. Neurosci..

[40] Demirel S.H., Çetinkaya S. (2014). Hypoxia-inducible factor-1: Physiological and pathological response to hypoxia of cell. Sakarya Med. J..

[41] Barranco R., Bonsignore A., Ventura F. (2021). Immunohistochemistry in postmortem diagnosis of acute cerebral hypoxia and ischemia: A systematic review. Medicine.

[42] Schuijt G. (1990). Iatrogenic fractures of ribs and vertebrae during delivery in perinatally dying calves: 235 cases (1978–1988). J. Am. Vet. Med. Assoc..

[43] Yildiz R., Aydogdu U., Guzelbektes H., Coskun A., Sen I. (2017). Venous lactate, pH and partial pressure of carbon dioxide levels as prognostic indicators in 110 premature calves with respiratory distress syndrome. Vet. Rec..

[44] Chawla D. (2020). Biomarkers for Prognostication in Hypoxic-Ischemic Encephalopathy. Indian J. Pediatr..

[45] Chen C., Qin H., Tan J., Hu Z., Zeng L. (2020). The Role of Ubiquitin-Proteasome Pathway and Autophagy-Lysosome Pathway in Cerebral Ischemia. Oxid. Med. Cell. Longev..

[46] Kecskes Z., Dunster K.R., Colditz P.B. (2005). NSE and S100 after hypoxia in the newborn pig. Pediatr. Res..

[47] Matuszczak E., Tylicka M., Komarowska M.D., Debek W., Hermanowicz A. (2020). Ubiquitin carboxy-terminal hydrolase L1—Physiology and pathology. Cell Biochem. Funct..

[48] Zeng S., Huang Y., Zhong T., Huang T., Dong X., Zhu H., Ouyang F. (2021). The expression and clinical value of ubiquitin carboxyl-terminal hydrolase L1 in the blood of neonates with hypoxic ischemic encephalopathy. Transl. Pediatr..

[49] Yang Z., Xu H., Sura L., Arja R.D., Patterson R.L., Rossignol C., Albayram M., Rajderkar D., Ghosh S., Wang K. (2022). Combined GFAP, NFL, Tau, and UCH-L1 panel increases prediction of outcomes in neonatal encephalopathy. Pediatr. Res..

[50] Gazzolo D., Vinesi P., Bartocci M., Geloso M.C., Bonacci W., Serra G., Haglid K.G., Michetti F. (1999). Elevated S100 blood level as an early indicator of intraventricular hemorrhage in preterm infants, correlation with cerebral Doppler velocimetry. J. Neurol. Sci..

[51] Nagdyman N., Kömen W., Ko H.K., Müller C., Obladen M. (2001). Early biochemical indicators of hypoxic-ischemic encephalopathy after birth asphyxia. Pediatr. Res..

[52] Santotoribio J.D., Cañavate-Solano C., Quintero-Prado R., González-Macías C., Soto-Pazos E., Vilar-Sanchez Á., Mesa-Suárez P., Ramos-Ramos V., Cuadros-Muñoz J.F., Mayor-Reyes M. (2019). Neuroapoptosis in newborns with respiratory acidosis at birth. Clin. Biochem..

[53] Fakher H., El-Shafey R., Diab A., Abdelmaksoud S., Abdel Raziq H. (2022). Lactate and S100 Protein as Early Biochemical Indicators of Birth Neonatal Asphyxia Caused by Intrauterine Umbilical Cord Strangulation: A Medicolegal View. Zagazig J. Forensic Med. Toxicol..

[54] Li F.J., Zheng S.R., Wang D.M. (2020). Adrenomedullin: An important participant in neurological diseases. Neural Regen. Res..

[55] Somay G., Halac G.U., Uslu E., Aydin S. (2007). Plasma adrenomedullin in acute ischemic stroke. Neurosciences.

[56] Zhang H., Tang B., Yin C.G., Chen Y., Meng Q.L., Jiang L., Wang W.P., Niu G.Z. (2014). Plasma adrenomedullin levels are associated with longterm outcomes of acute ischemic stroke. Peptides.

[57] Serrano-Ponz M., Rodrigo-Gasque C., Siles E., Martinez-Lara E., Ochoa-Callejero L., Martinez A. (2016). Temporal profiles of blood pressure, circulating nitric oxide, and adrenomedullin as predictors of clinical outcome in acute ischemic stroke patients. Mol. Med. Rep..

[58] Mitome-Mishima Y., Miyamoto N., Tanaka R., Shimosawa T., Oishi H., Arai H., Hattori N., Urabe T. (2014). Adrenomedullin deficiency and aging exacerbate ischemic white matter injury after prolonged cerebral hypoperfusion in mice. BioMed Res. Int..

[59] Mahmoodazdeh A., Shafiee S.M., Sisakht M., Khoshdel Z., Takhshid M.A. (2020). Adrenomedullin protects rat dorsal root ganglion neurons against doxorubicin-induced toxicity by ameliorating oxidative stress. Iran. J. Basic Med. Sci..

[60] Ferriero D.M. (2004). Neonatal brain injury. N. Engl. J. Med..

[61] Buonocore G., Groenendaal F. (2007). Anti-oxidant strategies. Semin. Fetal Neonatal Med..

[62] Florio P., Perrone S., Luisi S., Vezzosi P., Longini M., Marzocchi B., Petraglia F., Buonocore G. (2006). Increased plasma concentrations of activin a predict intraventricular hemorrhage in preterm newborns. Clin. Chem..

[63] O’Sullivan M.P., Denihan N., Sikora K., Finder M., Ahearne C., Clarke G., Hallberg B., Boylan G.B., Murray D.M. (2021). Activin A and Acvr2b mRNA from Umblical Cord Blood Are Not Reliable Markers of Mild or Moderate Neonatal Hypoxic-Ischemic Encephalopathy. Neuropediatrics.

[64] Su X., Huang L., Xiao D., Qu Y., Mu D. (2018). Research Progress on the Role and Mechanism of Action of Activin A in Brain Injury. Front. Neurosci..

[65] Florio P., Abella R., Marinoni E., Di Iorio R., Li Volti G., Galvano F., Pongiglione G., Frigiola A., Pinzauti S., Petraglia F. (2010). Biochemical markers of perinatal brain damage. Front. Biosci..

[66] Zaigham M., Lundberg F., Hayes R., Undén J., Olofsson P. (2016). Umbilical cord blood concentrations of ubiquitin carboxy-terminal hydrolase L1 (UCH-L1) and glial fibrillary acidic protein (GFAP) in neonates developing hypoxic-ischemic encephalopathy. J. Matern. Neonatal Med..

[67] Noorishakdam M., Savabieh S., Sahirifi M.E. (2020). Biomarkers of hypoxic-ischemia encephalopathy in newborns. World J. Peri Neonatol..

[68] Wals P., Jedeiken R., Ellis G., Primhak R., Makela S.K. (1982). Assessment of neurologic outcome in asphyxiadet term infants by used of serial CK-BB isoenzymes measurement. J. Pediatr..

[69] Becker M., Menzel K. (1978). Brain-typical creatine kinase in the serum of newborn infants with perinatal brain damage. Acta Pediatr. Scand..

