# Supplementary material for: The Usefulness of Serum Brain Damage Biomarkers in Detection and Evaluation of Hypoxic Ischemic Encephalopathy in Calves with Perinatal Asphyxia
Source: Animals (Basel). 2022 Nov 21;12(22):3223. doi: 10.3390/ani12223223 (PMC9686605; doi:10.3390/ani12223223)
Supplement: Supplementary file 1 [file animals-12-03223-s001.zip › animals-2018649-supplementary.pdf]

Supplementary materials

Supplementary Table S1. Hemogram variables of healthy and calves with perinatal asphyxia.

Variable		Time of evaluation (hours)			
		Admission (n_H:10, n_A:25)	24 (n_H:10, n_A:12)	48 (n_H:10, n_A:12)	72 (n_H:10, n_A:12)
WBC (m/mm^3)	Healthy	8.75 (5.44-13.04)	6.10 (4.23-9.12)	5.39 (4.46-15.97)	5.80 (4.74-11.40)
	Asphyxia	10.05 (1.90-42.84)	8.10 (4.99-9.70)	9.78 (3.24-48.24)	8.69 (5.05-23.64)
	P value				
RBC (m/mm^3)	Healthy	7.68 (7.06-9.80)	7.39 (6.71-8.36)	7.71 (5.26-9.42)	7.76 (5.17-8.68)
	Asphyxia	7.91 (4.34-12.83)	7.35 (5.55-7.69)	7.50 (4.91-9.90)	6.91 (5.75-8.96)
	P value				
HCT (%)	Healthy	33.15 (14.4-49.70)	29.60 (27.50-39.60)	30.40 (21.40-37.80)	32.35 (19.90-35.00)
	Asphyxia	33.50 (15.40-64.20)	30.60 (22.80-35.20)	34.90 (14.70-40.70)	31.30 (21.50-36.10)
	P value				
Hb (g/dL)	Healthy	9.15 (7.50-13.20)	9.90 (6.90-13.00)	10.00 (5.60-12.20)	9.55 (6.60-11.50)
	Asphyxia	10.60 (2.50-14.40)	7.10 (4.00-8.50)	9.20 (5.40-13.00)	7.25 (6.40-8.10)
	P value				
THR (m/mm^3)	Healthy	189.00 (200-310)	184.50 (172-247)	204.00 (143-384)	295.50 (165-385)
	Asphyxia	172.00 (85-424)	131.00 (131-243)	182 (123-404)	175.00 (141-285)
	P value				

n_H: number of calves included in the healthy group, n_A: number of calves included in the asphyxia group, WBC (Total leukocyte), RBC (erythrocyte), HCT (hematocrit), Hb (hemoglobin), THR (thrombocyte).

Supplementary Table S2. Scores of histopatholgical lesions in nonsurvivo calves with perinatal asphyxia.

Animal no.	INC	NF	Gliosis	MCI	Hyperemia	ECS	Edema	Hemorrhage	Total
1	2	2	2	2	4	2	2	2	18
2	1	1	2	1	4	1	2	0	13
3	3	3	3	0	2	1	3	2	17
4	2	2	3	1	2	1	2	0	13
5	3	3	3	2	3	2	3	2	21
6	3	3	3	1	4	3	3	2	22
7	3	4	4	1	4	3	3	1	23
8	1	2	2	0	3	1	2	1	12
9	2	3	2	0	2	1	2	0	12
10	1	2	2	0	4	2	3	1	15
11	3	2	3	0	3	1	2	1	15
12	1	2	2	0	2	1	2	0	10
13	2	3	2	0	2	1	2	0	12

Ischemic neuronal changes (INc); Neuronophagia (NF); Mononuclear cell infiltration (MCI); Endothelial cell swelling (ECS).

Supplementary Table S3. Immunohistochemistry scores in nonsurvivor calves with perinatal asphyxia.

Animal no.	IHC score
1	2
2	3
3	1
4	2
5	2
6	1
7	2
8	0
9	1
10	3
11	2
12	1
13	1
Average	1.61
